# The crystal structure of MreC provides insights into polymer formation

**DOI:** 10.1002/2211-5463.13296

**Published:** 2022-01-07

**Authors:** Qin Xu, Ning Sun, Qingjie Xiao, Chia‐ying Huang, Mengxue Xu, Weizhe Zhang, Lina Li, Qisheng Wang, Vincent Olieric, Weiwu Wang, Jianhua He, Bo Sun

**Affiliations:** ^1^ Shanghai Institute of Applied Physics Chinese Academy of Sciences Shanghai China; ^2^ University of Chinese Academy of Sciences Beijing China; ^3^ Shanghai Advanced Research Institute Chinese Academy of Sciences Shanghai China; ^4^ Department of Microbiology, College of Life Sciences Nanjing Agricultural University China; ^5^ Swiss Light Source Paul Scherrer Institute Villigen‐PSI Switzerland

**Keywords:** cell shape‐determining protein, crystal structure, filament‐like structure, MreC, polymer formation

## Abstract

MreC is a scaffold protein required for cell shape determination through interactions with proteins related to cell wall synthesis. Here, we determined the crystal structure of the major periplasmic part of MreC from *Escherichia coli* at 2.1 Å resolution. The periplasmic part of MreC contains a coiled‐coil domain and two six‐stranded barrel domains. The coiled‐coil domain is essential for dimer formation, and the two monomers are prone to relative motion that is related to the small interface of β‐barrel domains. In addition, MreC forms an antiparallel filament‐like structure along the coiled‐coil direction, which is different from the helical array structure in *Pseudomonas aeruginosa*. Our structure deepens our understanding of polymer formation of MreC.

## Graphical abstract

As a scaffolding protein, MreC is required to interact with proteins associated with cell wall synthesis to determine the cell shape. The crystal structure of MreC shows that the peripheral portion of MreC contains a coiled‐coil and two six‐stranded barrel‐like structural domains. In the resolved crystal structure, MreC exists as a dimer, further forms a tetramer, and eventually forms an antiparallel filamentous structure along the direction of the coiled coil. Our structure provides new structural insights and deepens the understanding of the polymer formation of MreC.

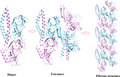

**Abbreviations**
MD: molecular dynamics simulation
PBP: penicillin‐binding protein
PDB: Protein Data Bank
SEC‐MALS: size exclusion chromatography with multiangle light scattering detection

Peptidoglycan, an important component of most bacterial cell walls, plays an important role in maintaining the shape of bacteria and resisting osmotic pressure [1]. Peptidoglycan is a multilayer network structure that is composed of glycan strands that are crosslinked by short peptides. The glycan strands are made by polymerization of *N*‐acetyl‐glucosamine and *N*‐acetylmuramic acid [2]. Peptidoglycan synthesis is under strict spatial and temporal control to ensure the normal growth and division of bacteria. Many antibiotics inhibit the growth of bacteria by disturbing the process of peptidoglycan synthesis [3, 4].

Several enzymes are directly involved in the synthesis of peptidoglycan. Glycosyltransferases catalyze the polymerization of the polysaccharide chain. Transpeptidases, also called penicillin‐binding proteins (PBPs), crosslink the short peptides between glycan strands [1, 4]. Besides, MreB, MreC and MreD help the localization of PBPs in *E. coli* and form a complex with specific PBPs to catalyze the peptidoglycan formation [5, 6]. MreB is the actin homologue that forms the cytoskeletal filaments to maintain the shape of bacterium [7, 8]. The two genes of *mreC* and *mreD* are located downstream of *mreB* in the same operon [9]. These three genes code for proteins that have been shown to maintain the rod‐like structure of cells, and the deletion of these genes leads to the formation of spherical cells and cell lysis in *E. coli* and *Bacillus subtilis* [5, 10, 11]. Previous studies have shown that MreC interacts with MreB, MreD and PBPs [5, 12, 13]. These proteins in *B. subtilis* have a similar trajectory around the cell [14]. Subcellular distribution of MreB in the cytoplasm depends on both MreC and MreD in *E. coli* [5]. MreC acts as a hub in complex formation. Besides, previous study also showed MreC in *Caulobacter crescentus* presents a helical or banded pattern [15]. Interestingly, MreC can maintain helical localization pattern in the periplasmic space, even when the compound A22 was used to disrupt the polymerization of MreB [15, 16]. Further, internal reflection fluorescence microscopy revealed that MreB and MreC form patches that showed circumferential motion [17]. Another study revealed that MreB can form the structure of antiparallel filaments *in vivo* [7]. Considering that MreC and MreB have similar distribution characteristics, and MreC can maintain the structural characteristics after disrupting the polymerization of MreB, it is suggested that MreC may also form a filament‐like structure.

The crystal structure of the major periplasmic part of MreC has been reported for some species [6, 18]. The complex crystal structure of MreC and PBP2 has also been determined for *Helicobacter pylori* and showed that MreC acts as a scaffold protein to regulate the synthesis of peptidoglycan [13]. Here, we determined the crystal structure of the major periplasmic domain of MreC from *E. coli* at 2.1 Å resolution, revealing an antiparallel filament‐like state that has not been reported yet.

## Materials and methods

### Expression and purification

The cDNA of encoding periplasmic part MreC (position of amino acid: 36–367) from *E. coli* BL21(DE3) strain was subcloned into Pet21b. *E. coli* BL21(DE3) cells carrying the MreC expression plasmid grow in LB medium at 37 °C, and MreC overexpression was induced by adding 0.4 mm isopropyl‐d‐thiogalactoside when absorbance (*A*_600_) reached between 0.6 and 0.8. The cells were further grown overnight at 22 °C and harvested by centrifugation. Cells were homogenized in lysis buffer [25 mm Tris–HCl (pH 8.0), 150 mm NaCl and 1 mm PMSF] and lysed using cell disruptor. The bacteria lysate was centrifuged at 13,800 *g* for 1 h. The supernatant was then loaded to equilibrated nickel‐nitrilotriacetic acid chromatography (Ni‐NTA, Qiagen, Germantown, MD, USA) resin twice. The resin was washed with wash buffer [25 mm Tris–HCl (pH 8.0), 150 mm NaCl, 20 mm imidazole]. The purpose protein was eluted with a solution of 25 mm Tris–HCl (pH 8.0), 150 mm NaCl and 300 mm imidazole. Eluted protein was then concentrated for further purification by gel filtration Superdex 200 Increase 10/300 GL column in 25 mm Tris–HCl (pH 8.0), 150 mm NaCl. The peak fractions were collected and snap frozen in liquid nitrogen and stored at −80 °C for crystallization.

MreC was crystalized at 18 °C using the sitting‐drop vapor diffusion method. The crystals appear in about 2 weeks, and the crystallization condition consisted of 9.9% Isopropanol, 9.9% (v/v) polyethylene glycol (PEG3350) and 0.1 M Tris–HCl (pH 8.5). All crystals were harvested and immediately snap‐frozen in liquid nitrogen after soaking in cryoprotectant that contains 25% glycerol.

### Data collection and structure determination

The datasets of MreC were collected at beamlines BL18U1 and processed with HKL3000 [19, 20]. The structure of MreC was solved by molecular replacement using Phaser [21] and MreC from *Listeria monocytogenes* Protein Data Bank (PDB: 2J5U) as the initial model. Major periplasmic domain of the protein shares 25% sequence identity with MreC of *E. coli*. The model of MreC was completed by using iterative refinement and model building by PHENIX [22, 23] and Coot [24]. The refinement statistics are summarized in Table 1.

**Table 1:** Data collection and refinement statistics. The value in parentheses is statistics parameter of the highest resolution shell. R_merge_ was defined by Diederichs and Karplus [29]. CC_1/2_ is the Pearson correlation coefficient of two half datasets as described by Karplus and Diederichs [30]. SSRF, Shanghai Synchrotron Radiation Facility.

Data	MreC
Integration package	HKL3000
Beamlines of SSRF	BL18U1
Space group	*C*_1_2_1_
Unit cell (Å)	133.3 47.6 77.3
Unit cell (°)	90 99.2 90
Wavelength (Å)	0.9793
Resolution (Å)	29.85 to 2.1 (2.14 to 2.1)
R_merge_ (%)	0.104 (0.474)
CC_1/2_	0.99 (0.70)
I/sigma	7.1 (1.3)
Completeness (%)	98.9 (94.0)
No. of measured reflections	83 679 (3403)
No. of unique reflections	27 880 (1309)
Redundancy	3
R_work_/R_free_ (%)	18.7/23.6
No. of atoms	3471
Protein	3285
Others	186
Average B value (Å^2^)	38.5
Protein	38.6
Others	37.7
rmsd	
Bonds (Å)	0.0059
Angle (°)	0.83
Ramachandran plot statistics (%)	
Most favorable	96.68
Allowed	3.32
Disallowed	0

### Size exclusion chromatography with multiangle light scattering detection

Size exclusion chromatography with multiangle light scattering detection (SEC‐MALS) experiments were carried out at 25 °C with multiangle light scattering detector (MALS; Wyatt Dawn Heleos‐II) and a size exclusion chromatography (Superdex 200 Increase 10/300 GL column) for measuring the relative molecular mass of protein. Protein samples (˜2 mg·mL^−1^, 100 μL) were injected in a buffer containing 25 mm Tris–HCl (pH 8.0) and 150 mm NaCl. ASTRA software (Wyatt Technology, Santa Barbara, CA, USA) was used to calculate the molecular weight of protein.

### Molecular dynamics simulation

Molecular dynamics simulations (MDs) were carried using GROMACS software with CHARMM36 force field (http://www.gromacs.org/) [25]. With periodic boundary conditions, the simulation was conducted in a cubic box with a dimension of 150 × 150 × 150 Å^3^. The minimum distance was set to 2.5 nm between the protein and edge of the box. The system was solvated with TIP3P for the water model and added with 150 mm NaCl. To relax the original structure, we used the steepest descent minimization method. After 12.5 ps NVT (constant number of particles, volume and temperature) and 125 ps NPT (constant number of particles, pressure and temperature) equilibrations, we positioned the restraints of the protein. The simulation was further run for 100 ns with 2‐fs timestep at temperature of 310 K and constant pressure (1 bar). During the MD process, the bond length was constrained by the LINCS algorithm [26]. The electrostatic interactions were calculated using the Particle Mesh Ewald method, with a cutoff of 10 Å for nonbonded interactions [27].

## Results

### Overall structure of MreC periplasmic domain

**Fig. 1:**
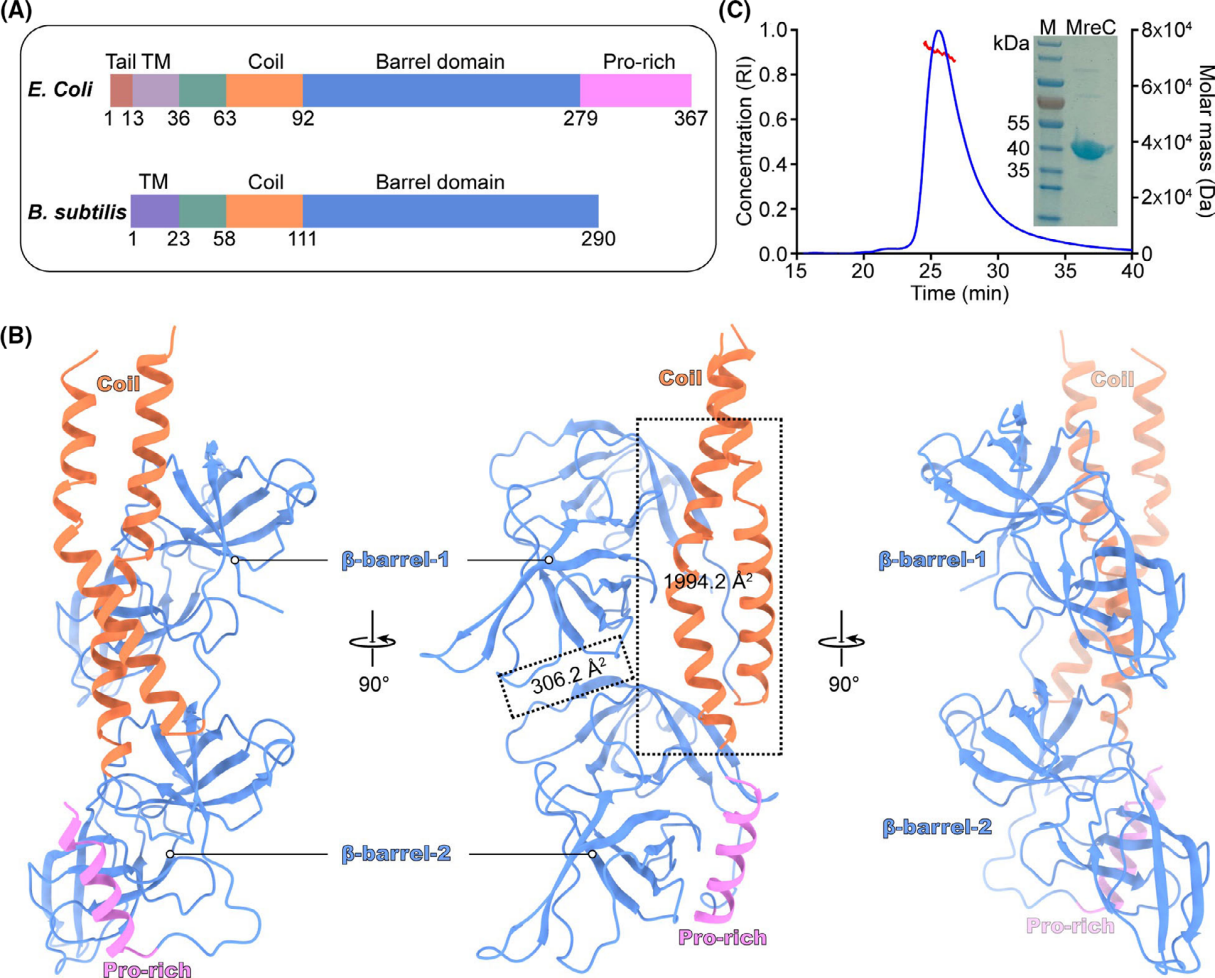
Crystal structure of MreC from *E. coli*. (A) Domain arrangement of MreC. (B) Overall structure of periplasmic domain for MreC in an asymmetric unit. The interaction interface was calculated via PDBePISA. (C) SEC‐MALS experiments. The calculated molar masses are indicated by the red line. Lane 1, protein marker; lane 2, purified MreC. Pro‐rich, compositional bias for proline; RI, refractive index; TM, transmembrane helix.

The full‐length MreC in *E. coli* contains a predicted transmembrane helix and periplasmic domain. The periplasmic domain is divided into a coiled coil, a β‐barrels domain and a pro‐rich region (Fig. 1A). We purified the truncated MreC (residues 36–367), which lacks the N‐terminal transmembrane region. The crystal of MreC diffracted to 2.1 Å resolution with space group C121 and unit cell *a* = 133.3 Å, *b* = 47.6 Å, *c* = 77.3 Å, *α* = γ = 90° and β = 99.2° (Table 1). The overall crystal structure shows a dimeric state of the periplasmic part of MreC in the asymmetric unit (Fig. 1B). Each monomer contains a long N‐terminal coil and two six‐stranded barrels. Two N‐terminal coils interweave with each other, and the coil of one copy inserts into the position between C‐terminal β‐barrels and the N‐terminal coil of another copy. The area of interface between the two monomers is 2214.3 Å^2^. The area of interface for coiled coil and the other monomer is 1994.2 Å^2^, while the area of the interface between the β‐barrel domain of the two monomers is only 306.2 Å^2^. This implies that the interface between β‐barrels of the two monomers is weak, and the coiled coil is important for maintaining the dimer structure. The molecular weight of the truncated MreC is about 37 kDa, while the result of SEC‐MALS indicated a molecular weight of about 73 kDa (Fig. 1C). These results suggested that MreC exists as a dimer in solution, which is consistent with the crystal structure (Fig. 1B).

**Fig. 2:**
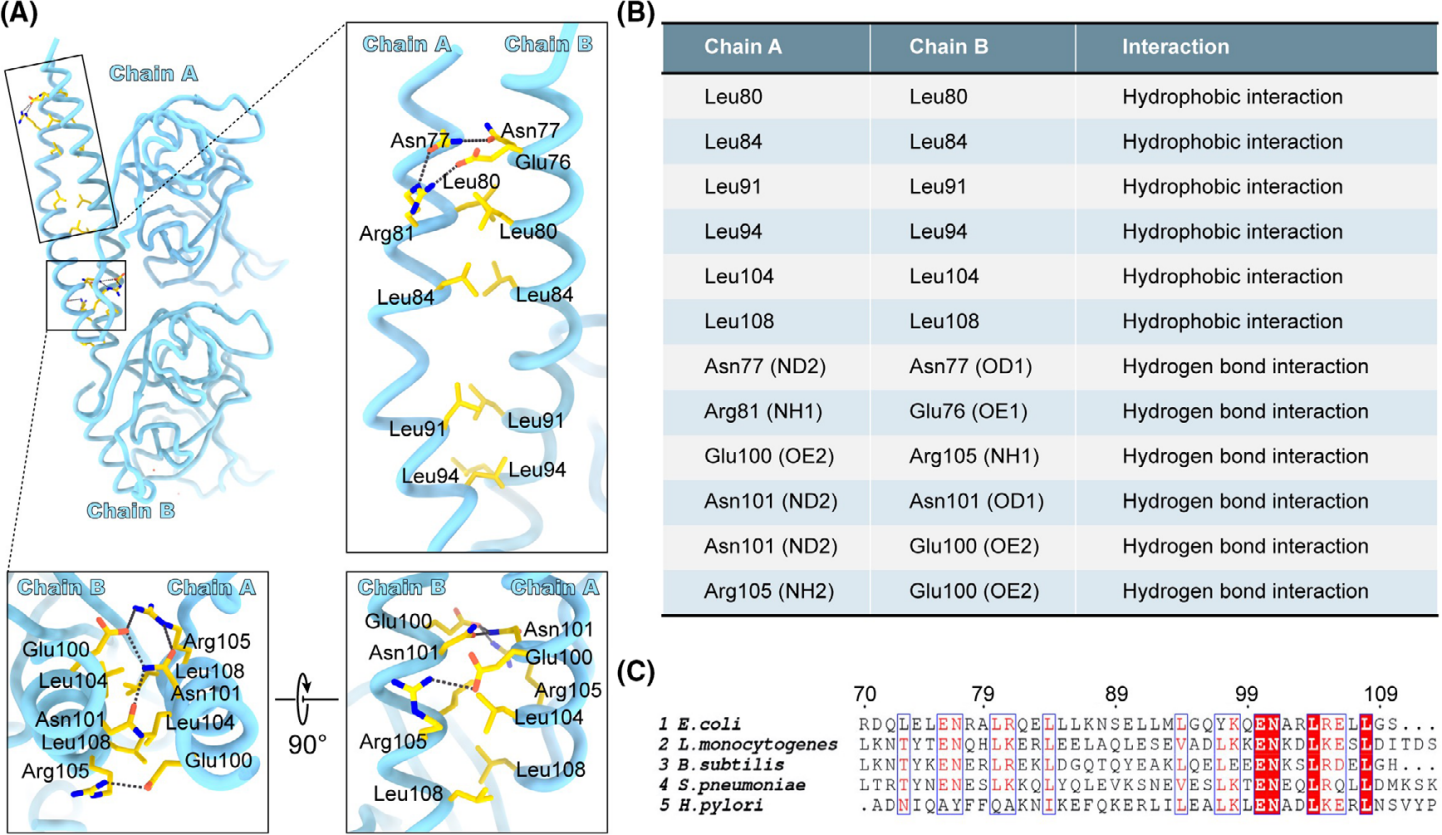
Interaction of coiled coil. (A) Residues that are involved in interaction are shown as stick‐and‐ball model. The hydrogen bonds were shown by black dashed line. The oxygen and nitrogen atoms are colored red and blue. (B) Table listed the residues of interaction for the coiled coil between chain A and chain B. (C) Alignment of coiled‐coil sequence from multiple bacteria.

Further analysis of the details of the interfaces showed that pairs of leucine establish a leucine zipper that maintains the interaction for two coiled coils (Fig. 2A). The pairs of leucine residues include Leu80, Leu84, Leu91, Leu94, Leu104 and Leu108. Besides, some polar residues provide hydrogen bond interactions (Fig. 2A). The residues of interaction for the coiled coils were listed in Fig. 2B. These residues are relatively conserved in bacteria (Fig. 2C), although MreC in different species has a lower sequence identity. We suggest that MreC in other species also forms a dimer by coiled‐coil interaction.

### Structure superposition of MreC from different species

**Fig. 3:**
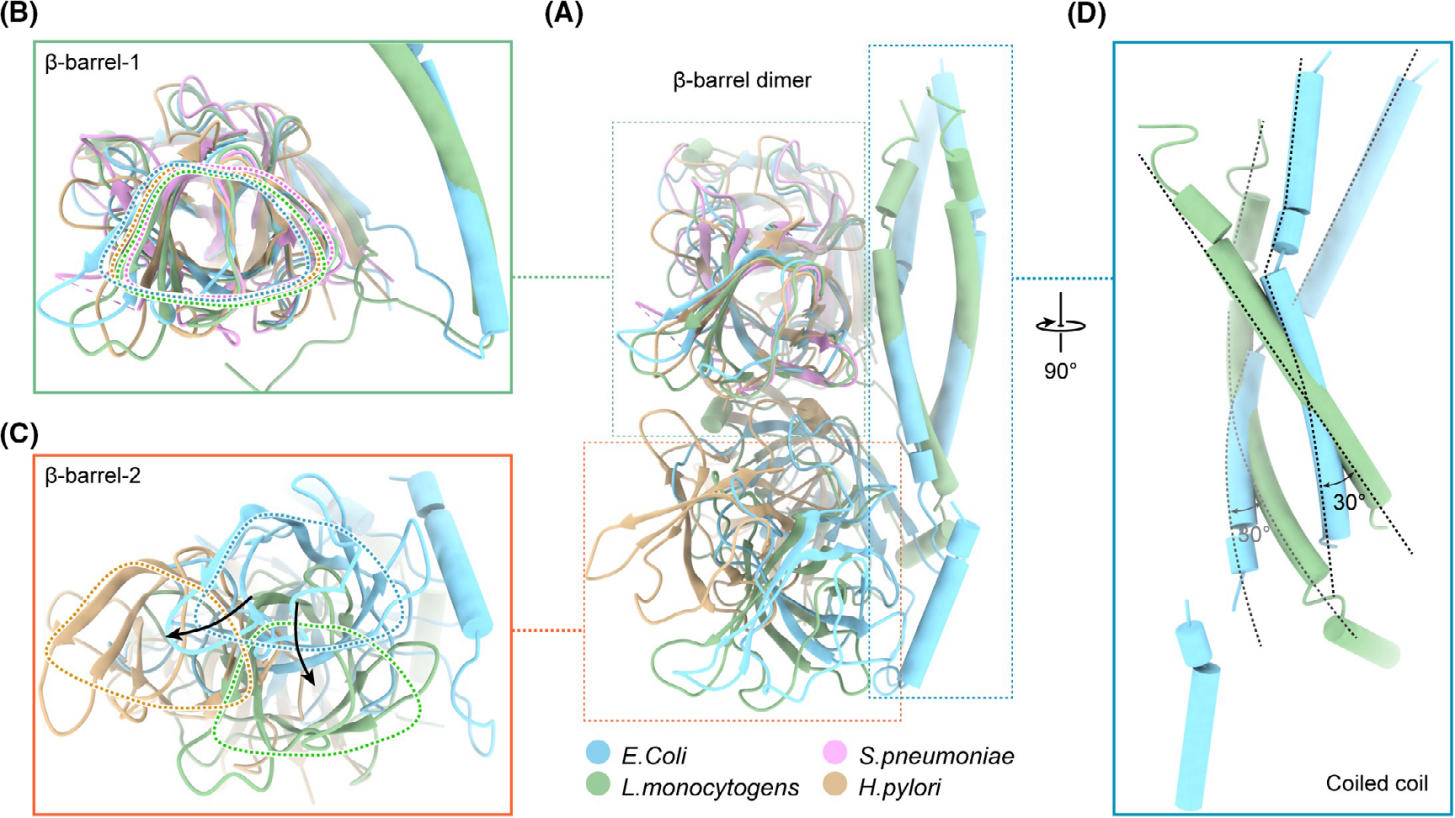
Structure comparison of MreC periplasmic domain from *E. coli*, *L. monocytogenes* (PDB: 2J5U), *S. pneumoniae* (PDB: 2QF5) and *H. pylori* (PDB: 5LP5). Rectangular region is the local enlarged view. The circle corresponds to the outline of β‐barrel domain. (A) Superposition of MreC periplasmic domain from *E. coli*, *L. monocytogenes* (PDB: 2J5U), *S. pneumoniae* (PDB: 2QF5) and *H. pylori* (PDB: 5LP5). Rectangular region is the local enlarged view for β‐barrel‐1 domain (B), β‐barrel‐2 domain (C) and coiled coil (D), respectively. The circle corresponds to the outline of β‐barrel domain.

**Fig. 4:**
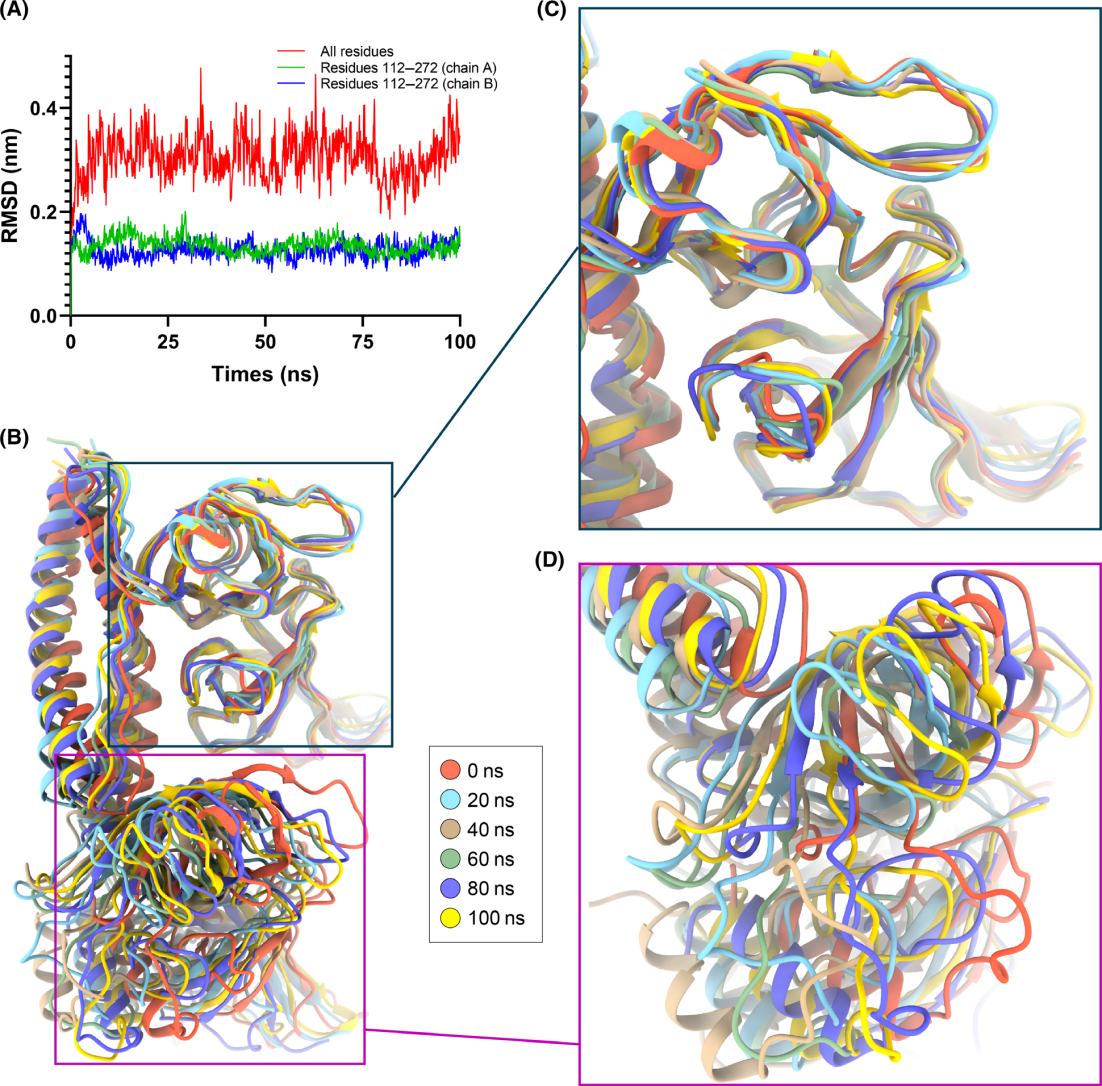
MD for the crystal structure of MreC. (A) rmsd values of Cα atom (with respect to the crystal structure) during 100‐ns MD simulations. Red line refers to rmsd values of the overall crystal structure, and blue line and green line refer to rmsd values for β‐barrels domain of A chain and B chain, respectively. (B) Snapshots are extracted every 20 ns, and β‐barrels domain of A chain is overlapped to observe the position change of two monomers. Rectangular region is the local enlarged view for β‐barrel domain of A chain (C) and β‐barrel domain of B chain (D), respectively.

Five structures of periplasmic MreC have been solved. MreC from *L. monocytogenes* contains the N‐terminal coiled coil, which forms an asymmetric dimer. Another two structures contain only the β‐barrels domain with Cα rmsd of 0.897 Å (*E. coli/L. monocytogenes*), 1.743 Å (*E. coli/S. pneumoniae*) and 5.13 Å (*E. coli/H. pylori*), respectively (Fig. 3A). Further, we compared the relative positions of the two monomers by superposing the β‐barrels domain of chain A for *E. coli*, *L. monocytogenes* and *H. pylori* (Fig. 3B,C). The packing of the two monomers is similar, but there is a deflection of about 30° for the coiled coil and about 10 Å movement for the β‐barrel domains between *E. coli* and *L. monocytogenes* (Fig. 3D). There is a movement of about 15 Å for the β‐barrel domains between *E. coli* and *H. pylori* (Fig. 3D). The interface between the β‐barrels of the two monomers is weak, which may suggest that the domains are easy to move relatively to each other. Further, MD was carried out for 100 ns to investigate the change for relative positions of the two monomers (Fig. 4A). The rmsd of Cα for the overall structure is about 4 Å in 100 ns. Interestingly, the rmsd of Cα is <1.5 Å for barrel domain of the two monomers (Fig. 4A), which suggest the barrel domain is relatively rigid. Snapshots captured from different times intuitively showed the obvious movement between the two monomers (Fig. 4B–D).

### Filament‐like form of MreC in the crystal

**Fig. 5:**
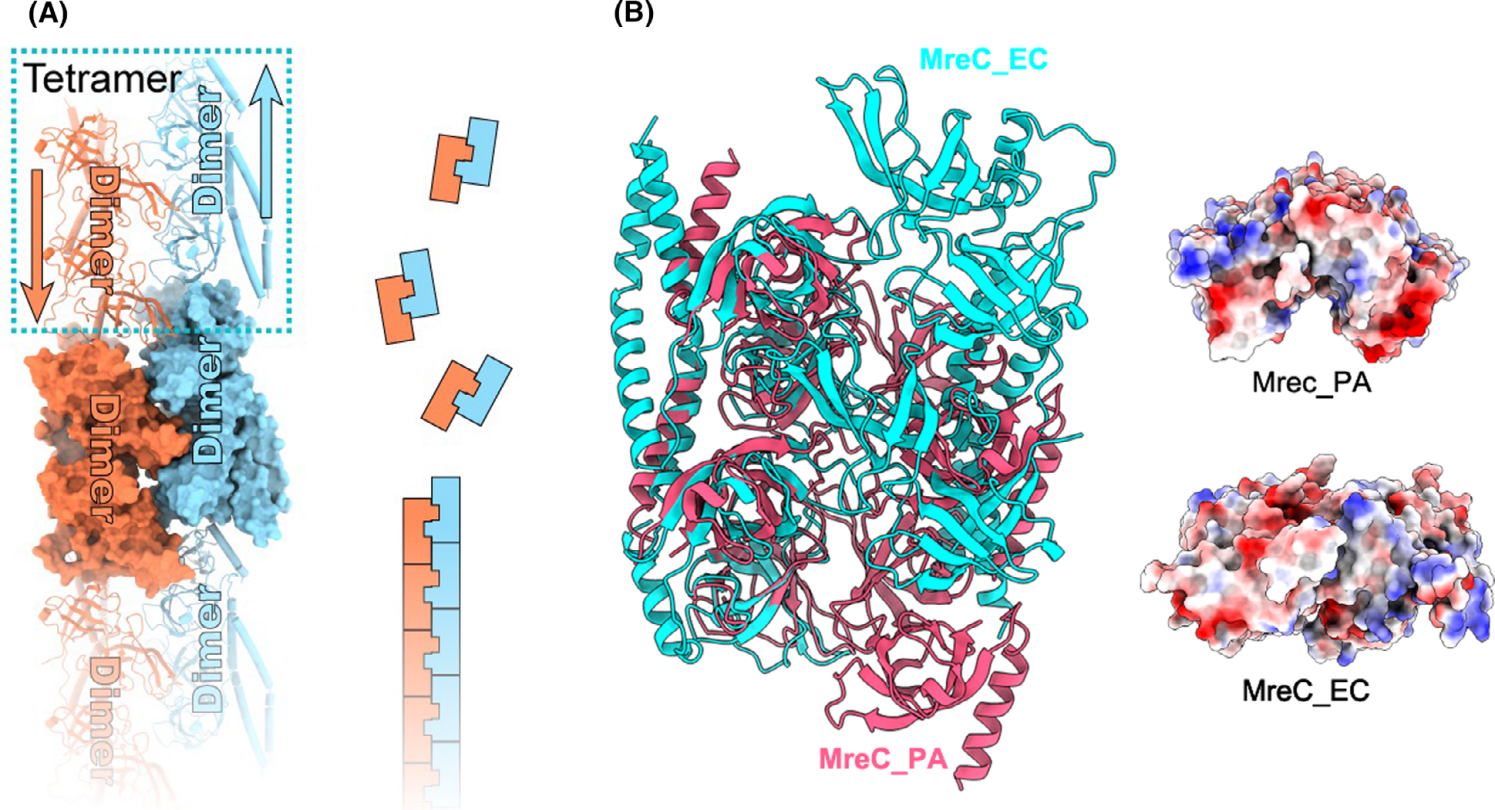
MreC packing to the state of antiparallel filament‐like. (A) Left is the packing of MreC with the *C*_1_2_1_ symmetry, and right is a model diagram for filament structure. (B) Left is a structure comparison of tetramer from *E. coli* and *P. aeruginosa*, and right is electrostatic surface potential of MreC that is viewed along the direction of coiled coil. EC, *E. coli*; PA, *P. aeruginosa*.

MreC solved from this study forms antiparallel double‐filaments‐like structure of head to tail in the crystal after generating the symmetry mate (Fig. 5A). The interaction for two adjacent filaments was formed by the two barrel‐shaped domains on the other side of the coil (Fig. 5A). The dimer coiled‐coil interface is only 291Å^2^, which is unlikely to maintain the filaments‐like structure on its own. The tetramer form provides a larger interface for the formation of the filament structure (Fig. 5A). The interface of junction is about 1125 Å^2^ as calculated by Proteins, Interfaces, Structures and Assemblies (PISA). The area of the interface is relatively large compared with MreB from *Caulobacter vibrioides* that also has been reported as forming filament with 844 Å^2^ at the junction interface. Besides, another group resolved recently the structure of helical array MreC (PDB: 6ZLV) from *P*. *aeruginosa* by electron microscopy with the tetramer as a unit [28]. Coiled‐coil and β‐barrels part of MreC from *E. coli* and *P. aeruginosa* share about 42% sequence identity, and the value of rmsd is about 0.85 Å over 135 Cα atoms of monomer and 2.8 Å over 296 Cα atoms of dimer. The tetramer state of MreC from *E. coli* and *P. aeruginosa* is formed by the interaction of β‐barrels part from two dimers with reversed orientations (Fig. 5B). Interestingly, an obvious difference is the tetramers from *P. aeruginosa* show a semicylinder associated with an angle of 135° between the two dimers, while the tetramers from *E. coli* show a plane surface (Fig. 5B).

## Discussion

Previous studies have shown that MreC forms a dimer through a coiled coil [5, 6]. The crystal structure reveals that the formation of the MreC dimer is related to the interaction of coiled coils, which contain a leucine repeat conserved among different species. This indicates that the MreC dimer is important to its function. Despite the local structure similarity of MreC with α‐lytic protease (PDB: 1QQ4) and the serine protease (PDB: 2AS9) [18], there is no evidence that MreC has catalytic functions. More evidence showed that MreC may act as a scaffold that can interact with various proteins to affect the spatial positioning of peptidoglycan synthesis. So far, five MreC structures from different species have been reported with sequence identity from 14% to 40% and share a similar three‐dimensional structure. Comparison of multiple structures and MD provides possible insights into the structural characteristics of MreC as dynamic and adaptable. Structure comparison of different species and MD showed that the small interface between β‐barrel domains is helpful to adjust the relative position of two monomers that is related to the interaction of various proteins for MreC. In turn, the coiled coil can help maintain the dimer state and stabilize the β‐barrel domain array. The array is conducive to the formation of tetramer by a larger interaction interface that is provided by the barrel‐like domain.

Besides, studies in vivo have suggested that MreC may form polymers, but it is not clear about this mechanism. The crystal structure of MreC in *E. coli* in this study reports the antiparallel filaments‐like form that is formed by an arrangement of tetramers as a unit. A recently released MreC structure (PDB: 6ZLV) presents polymers that are a helical arrangement in a tetramer unit. Considering the obvious difference of conformation in the tetramers between MreCs from *E. coli* and *P. aeruginosa*, we speculate that tetramer conformation is related to the polymer state of the helical arrangement and filament‐like. The structure provides an understanding of the mechanism of polymers formation.

## Conflict of interest

The authors declare no conflict of interest.

## Author contributions

JH, BS, QX and QX conceived and designed the experiments. QX and NS purified and crystallized protein. BS, QX, MX, WZ and LL collected the diffraction datasets and solved the structures. QX carried out MDs. JH, WW and BS wrote the manuscript. VO, CH and QW modified the paper. BS, JH and WW supervised the research.

## Data Availability

The structure factors and atomic coordinates of the MreC crystal structure are deposited in the PDB under accession number 7EFT.
